# The importance of histopathologic review of biopsies in patients with prostate cancer referred to a tertiary uro - oncology center

**DOI:** 10.1590/S1677-5538.IBJU.2018.0099

**Published:** 2019

**Authors:** Wagner Eduardo Matheus, Ubirajara Ferreira, Elimilson A. Brandão, Aline A. Ferruccio, Athanase Billis

**Affiliations:** 1 Departamento de Cirurgia, Hospital das Clínicas da Universidade Estadual de Campinas - UNICAMP, Campinas, SP, Brasil;; 2 Departamento de Urologia da Universidade Estadual de Campinas - UNICAMP, Campinas, SP, Brasil;; 3 Departamento de Urooncologia da Universidade Estadual de Campinas - UNICAMP, Campinas, SP, Brasil;; 4 Pontifícia Universidade Católica de Campinas - PUC, Campus II, Campinas, SP, Brasil;; 5 Departamento de Anatomia Patológica da Universidade Estadual de Campinas - UNICAMP, Campinas, SP, Brasil

**Keywords:** Prostatic Neoplasms, Neoplasm Grading, Pathology

## Abstract

**Introduction:**

In view of the detailed histologic evaluation of prostate cancer (PC), it is usually advisable to provide a “second opinion” to confirm diagnosis. This study aimed to compare the Gleason score (GS) of initial diagnosis versus that of histopathologic review of patients with PC. The secondary objective was to compare initial GS versus histopathologic review versus post - surgical histopathology.

**Material and methods:**

Retrospective study based on chart review of patients with PC that attended the Uro - oncology Department of Hospital das Clínicas - UNICAMP - Campinas, Brazil, from April, 2002, to April, 2012. Data were divided in groups: patients with biopsies performed elsewhere, biopsies after pathological review and histopathological results following retropubic radical prostatectomy (RRP). These were evaluated in relation to GS difference using Fleis's Kappa concordance coefficient.

**Results:**

402 PC patients, with a median age of 66 years, were evaluated. Reviewed GS showed worsening, with accuracy of 61.2%, and Kappa concordance value = 0.466. Among 143 patients submitted to surgery, GS varied widely, regarding initial evaluation, review and post - surgical RRP. Joint concordance of evaluations was weak (Kappa = 0.216), mainly due to almost no existence concordance between initial evaluation and following RRP (Kappa = 0.041).

**Conclusion:**

There is a great histopathological variation of initial GS versus reviewed GS. There is also a better correlation of reviewed GS and post - surgical GS than with initial GS. The second opinion by an uropathologist improves diagnosis and should be advised for better therapeutic decision.

## INTRODUCTION

Increase in life expectancy and determination of prostatic specific antigen (PSA) elevated the incidence of PC in the last decades. Prognosis is determined by histologic grade, PSA and digital rectum exam, of which GS is the most important to determine the best treatment for specific - risk groups (1-3).

Since an accurate histologic evaluation is mandatory, in many occasions it is necessary a “second opinion” to confirm the diagnosis and to determine the GS, and occasionally, the analysis by an uro - pathologist is decisive (4-6). Its recommendation is increasing due to its benefits, such as more efficient therapies for different tumors, lowering costs using correct treatments, and lower risks of legal exposure of the physician, for example (7).

At the uro - oncology ambulatory of Hospital das Clinicas - UNICAMP, all external histopathologic exams of PC are routinely revised. Each patient of that ambulatory with initial external diagnosis of PC provides the material for confirmation by the Pathology Department, before a new biopsy. The primary goal of this study was to compare initial diagnostic GS versus histologic review of patients with PC referred to our tertiary center. The second objective was to compare initial diagnostic GS versus post - surgical exams of patients submitted to RRP.

## MATERIALS AND METHODS

This is a retrospective study exclusively based on the review of 402 charts of patients with PC attended at the uro - oncology Department of HC - UNICAMP from April 2002 to April 2012.

The following data were collected: age, initial total PSA and after review, digital rectum exam, histopathologic findings, in special GS, D’Amico risk grade and treatments.

In 2005, during the analyzed period, the International Society of Urologic Pathology published modifications of the original system of PC grading. More restrict criteria were stablished for Gleason 3 classification, lowering its incidence in the pathological exams. Also, a secondary pattern was determined as worse prognosis observed at the needle biopsy. Since the present study was performed in a tertiary uro - oncology referral center, the pathology department adjusted to the new criteria, as soon as they were published in scientific literature. Therefore, charts analyzed after 2005 adopted the GS changes proposed by ISUP (8). Data were divided in groups, according to histologic exam momentum: Group 1 (external initial analysis) - initial results of external prostate biopsies, performed at non - tertiary clinics and hospitals, of patients referred to the ambulatory of uro - oncology; Group 2 (histopathologic review) - results obtained at the initial prostatic biopsies, using the same material (lamina and blocks) brought by the patients from the origin clinics, that were reviewed by a pathologist member of the Department of Pathology of our tertiary center; Group 3 (after RRP) - results of the pathological exam of the surgical block removed after RRP only of patients submitted to surgical treatment in the same tertiary uro - oncology service. Following data collection, groups were compared in relation to GS differences.

Inclusion criteria: patients with 40 to 80 years old, with PC diagnosis and previous biopsy elsewhere, that were referred to the uro - oncology ambulatory and were reviewed before definitive treatment.

Exclusion criteria: patients with diagnosis and initial prostatic biopsy provided by that tertiary center that did not need histopathologic review. Also, patients with lack of histologic exams, histopathologic findings or relevant clinical information were also excluded; 75% of data were considered sufficient. Data were analyzed descriptively and initially in an Excel chart and posteriorly were analyzed by SPSS software. Demographic data are presented in number and percentage. GS is showed in tables and frequency graphics.

Fleis's Kappa concordance coefficient of GS were calculated in different groups to verify initial biopsy concordance (Group-1), second opinion (Group-2) and after surgery (Group-3). Kappa values vary from – 1 to + 1. The higher the Kappa value, the stronger is the concordance of the analyzed groups. Kappa ± 1 refers to perfect concordance, Kappa = 0 refers to random concordance and Kappa values < 0 refer to low concordance, lower than expected randomly. Values ≥ 0.75 refer to good concordance (9).

## RESULTS

Median age of the 402 patients was 66 years and most showed initial and reviewed PSA ≤ 10 ng / dL (Table-1). Most used treatments were radiotherapy and RRP (19% and 17.5% respectively) followed by bilateral orquiectomy and hormone therapy (7.8% and 7%).

**Table 1:** Patients characteristics.

Variables		Frequency	%
**Age (year)**			
	40 to 49	14	3.5
	50 to 59	76	19
	60 to 69	181	45
	70 to 80	131	32.5
Medium (SD)		65.4 (8.4)	66 (40 – 80)
Median (min - max)			
		
**Initial PSA**			
	≤ 10	173	43
	> 10 and ≤ 20	129	32
	> 20	100	25
Medium (SD)		18.6 (47.8)	12 (3 – 544)
Median (min - max)			
		
**Review PSA**			
	≤ 10	168	42
	> 10 and ≤ 20	99	24.5
	> 20	135	33.5
Medium (SD)		21.5 (47.4)	12.5 (3.3 – 545)
Median (min - max)			

**Figure 1:**
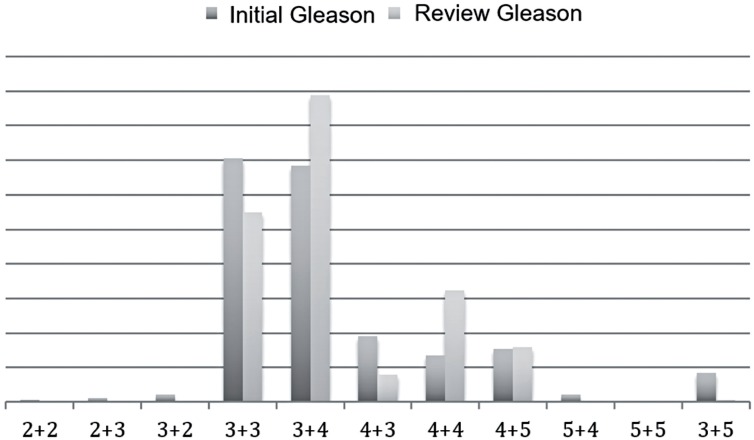
Initial and post - review GS of all patients.

When the 402 patients were analyzed, initial GS and after review varied, with a worsening of histologic classification (Table-2 / Figure-1). Accuracy between initial GS and revision was only 61.2% with Kappa = 0.466 and CI 95% (0.427 – 0.495).

**Table 2:** Initial and post - review GS of all patients.

Gleason	Initial		Review	
	Frequency	%	Frequency	%
2 + 2	1	0.3	0	0
2 + 3	2	0.5	0	0
3 + 2	4	1	0	0
3 + 3	141	35	110	27.4
3 + 4	137	34	178	44.3
4 + 3	38	9.5	16	4
4 + 4	27	6.7	65	16.2
4 + 5	31	7.7	32	8
5 + 4	4	1	0	0
5 + 5	0	0	0	0
3 + 5	17	4.3	1	0.2
**Total**	**402**	**100**	**402**	**100**

**Figure 2:**
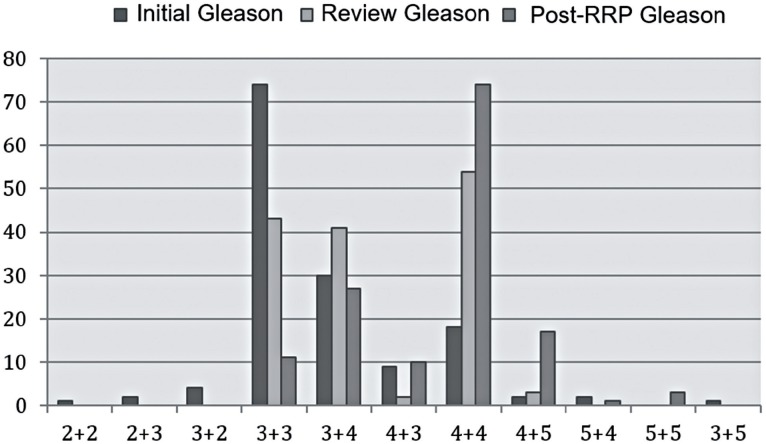
Initial, post - review and after RRP GS only of patients submitted to surgery.

Considering only the 143 patients submitted to surgery, GS varied widely from initial evaluation, review and after RRP (Table-3 / Figure-2). Accuracy and concordance values between initial GS and after RRP were the lowest among all theee comparisons: initial versus review, initial versus after RRP and review versus after RRP, with estimate 21% and – 0.041, respectively. Cojoint concordance of three evaluations was considered weak (Kappa = 0.216) mainly due to the almost inexistent accordance between initial and RRP (Kappa = 0.041).

**Table 3:** Initial and post - review GS and after RRP only of patients submitted to surgery.

Gleason	Initial		Review		Post - RRP	
	Frequency	%	Frequency	%	Frequency	%
2 + 2	1	0.7	0	0.0	0	0.0
2 + 3	2	1.4	0	0.0	0	0.0
3 + 2	4	2.8	0	0.0	0	0.0
3 + 3	74	51.7	43	30.1	11	7.7
3 + 4	30	21.0	41	28.7	27	18.9
4 + 3	9	6.3	2	1.4	10	7.0
4 + 4	18	12.6	54	37.8	74	51.7
4 + 5	2	1.4	3	2.1	17	11.9
5 + 4	2	1.4	0	0.0	1	0.7
5 + 5	0	0.0	0	0.0	3	2.1
3 + 5	1	0.7	0	0.0	0	0.0
**Total**	**143**	**100**	**143**	**100**	**143**	**100**

## DISCUSSION

Several studies have shown the importance of a second opinion by experienced uro - onco - pathologists, in order to obtain an accurate PC diagnosis (10, 11). Histological review may propose a more precise treatment of patients: from watchful waiting or active surveillance, to invasive therapies such as surgery, radiotherapy and hormone - therapy.

In this study, the degree of discrepancy of initial GS following review was close to 45%. Many causes may explain this alteration: lack of familiarity of non - tertiary hospital pathologists with the analyzed samples, difficulty to evaluate biopsies with Gleason score 4 and 5 after the change of classification proposed in 2005, and the bias due to the review performed by the same team that analyzed the histologic material following RRP. Also, the size of the biopsy samples, number of positive samples and percentage of cancer in each positive sample, since they were not individualized in a different vial, may also alter the final diagnosis and GS, preventing a better analysis on the place the sample was originally from (12). Also, Epstein and cols observed that 5% of needle biopsies described atypical glands suspected of carcinoma with a higher probability of change after expert review (4).

One of the first studies on second histopathologic opinion of PC was published by Epstein in 1996. After reviewing 535 patients, 7 (1.3%) were reclassified as benign, avoiding subsequent treatments, with significant savings, better quality of life, absence of collateral effects of surgery and radiotherapy and the possible reclassification to minimum tumor volume with indication to watchful waiting (13).

In 2001, Murphy and cols (7) reviewed 150 patients and observed 29 discrepancies in diagnostic interpretation and 14 resulted in change of treatment. The author was unaware if second opinion was an error of interpretation or a true difference of opinion, but concluded that treatment of patients was affected and therefore the review should be ordered as part of a complete evaluation.

Wayment and cols (11) in 2011 evaluated the use of second opinion in patients with urological tumors. Among 264 patients, 213 had material for reanalysis, being 117 patients with PC (55%), 83 with bladder cancer (39%), 5 with testicle tumor (2%), 5 with pelvis or ureteral tumor (2%), 2 retroperitoneal tumors (1%) and 1 renal tumor (0.5%). In 22 patients, it was observed disagreement of initial and reviewed diagnosis (10.3%), being 18 classified as important and 4 with lower importance, reinforcing the role of histopathologic review by uro - pathologists not only of PC but of all urologic tumors (7).

Berg et al. (14) stated that PC histopathology is closely associated to variability among different professionals. In their study, they analyzed prostatic biopsies review and compared initial reports, reviewed reports and RRP results in 350 patients.

For Berg et al. (14), PC histopathology is significantly associated to variability among different observers. In their study, they analyzed reviews of prostatic biopsies compared to initial biopsies reports, and RPP material in 350 patients. GS accordance between initial report and reviewed was 76.9%. Reviewed tumors had higher GS grades in 25% of patients, when primary GS = 6. Tumors were under-classified in 3% and 10.3% of patients with primary GS = 7 and ≥ 8, respectively. Also, in that study, there was a significant tendency to higher concordance between reviewed GS and surgical sample following RRP, which was also observed in our study.

Cury et al. (15) stressed that exact determination of GS at prostate biopsy is crucial to choose the correct treatment of PC, particularly of well - differentiated tumors, where imprecision may result in a very conservative treatment.

In our study, comparing analysis of GS by general pathologists and by tertiary centers uropathologists, it was possible to find a frequency of 34% of Gleason 3 + 3 determined by general pathologists and a drop to 27.4% of that GS by uropathologists analysis. It was also observed a significant increase of GS 3 + 4 (from 34% to 44.3%), reduction of Gleason 4 + 3 (from 9.5% to 4%) and a considerable increase of Gleason 4 + 4 (from 6.8% to 16.2%), respectively. These results are in agreement with the literature data, showing that the review by uropathologists or experienced pathologists in urological diseases usually increases GS value. When this analysis was performed only in patients submitted to RPP, the number of patients with GS 3 + 3 significantly reduced from 51.7% to 7.7%. On the other hand, GS 4 + 4 increased from 12.6% to 51.7%, respectively.

Even considering the study limitations, in special retrospective observation bias and GS alteration that alter homogeneity of information, the results confirmed the importance of review for better treatment of patients, as well as the need to improve the reproducibility of biopsy results, by continued education and specific training of pathologists.

## CONCLUSIONS

In this study, histopathologic review showed great histopathologic variation of initial GS versus reviewed. It also showed better correlation of reviewed classification with surgical sample than with initial GS. Therefore, it is concluded that uropathologist expert review is important for precise diagnosis and correct treatment, that must whenever possible be recommended before therapeutic decision.

## ABBREVIATIONS

PC: = Prostate cancer
GS: = Gleason Score
RRP: = Retropubic radical prostatectomy
PSA: = Prostatic specific antigen
